# A Unique Case

**Published:** 1886-07

**Authors:** W. H. Calfee

**Affiliations:** Waco, Texas


### A UNIQUE CASE.

*By W. H. Calfee, A. M., M. D., Waco, Texas.*

(For Daniel's Texas Medical Journal.)

IN a copy of your Journal for March, Dr. Paine, of Comanche, reports "A Rare Case," which recalls a unique case in my practice in December, 1885, in Waco. We were called to see Mrs. ——— at 2 o'clock p. m., who had been in labor several hours, in care of a midwife. At 4 o'clock, labor was progressing, and the child was delivered at 8 o'clock p. m., without any unusual conditions, except the cord was drawn so tightly around the child's neck we could not remove it by ordinary means till after delivery. The mother did well in her recovery.

The child's head and body were well developed, but both arms were stumps, with no forearms or elbows, and about the same length—two or three inches of humerus, whose distal extremities were well rounded with soft tissue, as though Dame Nature had done her work well; excepting the right arm, which had a small, nonligamentous appendage about one inch long—teat-like form. The legs above the knees were well developed, but both knees, or joints, only had partial motion—the right knee less than the left one. The legs below the knees were diminutive, as well as the feet, and were not more than half the usual size. The legs were short below the knees—about half the usual length—and corresponded. The feet, each, had but two toes—the great toe and the next larger, and were about half the usual length. There was talipes—calcaneus—of the right foot, and the second or smaller toe on left foot was ligamentous and muscular.

After ten days time we amputated the little appendage on the right arm, and found it contained a small artery, which we ligated. For the calcaneus we practiced sub-cutaneous tenotomy of the tebialis anticus and the extensor proprius policis muscles, which brought the foot down readily ; then applied a compress and bandage.

The development of the smaller toe (the second in size) on the left foot, was such that it could not be of any service, and was a deformity. We amputated it, with slight hemorrhage. From the operations it readily recovered. At this time the child is healthy and growing.

We claim for the "freak of nature" a case of unique deformity.

### A CASE IN OBSTETRICS.

*By O. Eastland, M. D., Wichita Falls, Texas.*

(For Daniel's Texas Medical Journal.)

MAY 12th, 1886. Mrs. F., aged thirty-six, of German birth, long resident of Texas, mother of five children, had a sudden gush of water, per vagina, while waltzing about the room; immediately lying down, it was followed by pain, and, in my absence from town, another physician was summoned. For an hour or two, as I am informed, irregular pains of more or less intensity, occurred: but, with no dilation of the os; these finally subsided spontaneously, and on the following day she walked about the house with a certain degree of comfort, occasionally disturbed by a spasmodic pain, especially noticeable about 6 p. m., each day.

Ten days subsequent to this first loss of water, there was an intensification of the pain, and again a quantity of *liquor amnii* was discharged, necessitating a change of clothing, etc. Being again summoned, I found irregular uterine contractions occurring, with some slight loss of water with each pain; os thin, but unyielding, lying far back toward the sacrum, in the vicinity of the left synchondrosis. I attempted reposition, with some little success, but found the partially "bearing down" pain still ineffectual; then, with some hope of increasing uterine action, I attempted Kristeller's "*Expressio* Foetus," in a mild form. This being ineffectual, I administered a good quantity of Quinia, with hopes of oxytoccic effect; but was disappointed, not only in this, but it failed in producing the ordinary phenomena of cinchonism, despite the fact of an idiosyncrasy on the part of patient that prevents me from using even the smallest therapeutic quantities under ordinary circumstances. All in vain—and the pains subsided, leaving the patient with a moderate degree of comfort, to which (since my efforts seemed unnecessary in provoking fœtal expulsion) I attempted to add by an anodyne. The day following she was a little nervous, but free from severe pain; there was considerable gastric disturbance, and much fœtal movement, especially upward. Subsiding, as she did, into a semi-comfortable state, without anything alarming, at the same time without anything encouraging as to an early terminatioh, I was content to watch attentively at intervals, until about noon, June 2d, when I was summoned, and found true pains occurring, and a footling presentation. The child, an eight-pound girl, was in the hands of the nurse at 3:30 p. m., the placenta coming away in about twenty minutes, when the patient expressed herself as feeling "better than ever before." After the lapse of an hour, I drove leisurely back to my office, having taken, as I *always* do, McClintock's precaution of careful examination of the pulse, with an additional precaution of my own, which I strenuously observe, that of leaving some Lig. Ergotæ Purificatus, with specific directions for its use, if necessary.

I had scarcely arrived at home and seated myself, when a messenger, in haste, came to inform me that Mrs. F. was "sinking," which I, naturally enough, conjectured meant *post partum* hemorrhage. Hastily arriving at the scene, I found that situation familiar to those acquainted with the perils of the lying-in chamber—blanched and speechless lay the unconscious patient I had so recently left feeling well and cheerful. The nurse—an intelligent one—had, on the first alarm, given the Ergot, at the time of dispatching the messenger, and had repeated the dose a few minutes afterward, to which I forthwith added another, using compression on the iliac arteries, easily accessible through the relaxed abdominal walls, kneading the flaccid uterus quite vigorously; a responding contraction soon staunched the flow of the life current, so nearly exhausted. Stimulants, etc., restored consciousness after a time, and in three hours the extreme prostration was over, and while naturally quite weak for a number of days, her "getting up" has been fair.

This case presents a point of interest in the fact of delivery not occurring for three weeks after the rupture of the membranes, during which time there was no really *grave* symptoms developed by the mother, nor was there indication of ill to the fœtus, as shown after delivery, either from *intra-uterine* pressure, lack of lubrication or nourishment.

The functions of the amniotic fluid are recognized as a mechanical protection of the fœtus, facilitating motion *in utero*, and the maintainance of equilibrium in temperature, with a proper moisture of the fœtal integument. Perhaps there will be a number of accoucheurs, under whose eyes these lines may come, who have seen the wrinkled, loose, dry skin of the infant delivered after the amniotic fluid had been away for a considerable period of time; but nothing of this was visible in this case, and, in search of a reason for this, we can but conclude that, after the escape of this important fluid, the organs or factors of its production immediately set about the production of a new supply. This rational conclusion naturally interests us in the subject of its origin—a question yet *sub judice*. The histological character of the amnion inclines recent investigators to conclude with certainty that, during the entire period of gestation, the amnion, at least, presides in its evolution. Consisting of two layers—the epithelial, next the fœtus, and the connective tissue on the exterior—it would seem most capacitated for active functions in the earlier month of gestation, when these cells are observed to be in best condition for activity; but granting the truth of their degeneration in the latter months, there always remain numerous irregular cells, well filled for the processes of transudation and osmosis from the chorion and uterine walls.

